# Blockage of *CX3CL1* Attenuates Platelet and Leukocyte Recruitment in Murine Hepatic I/R

**DOI:** 10.1159/000524024

**Published:** 2022-03-11

**Authors:** Dominik Funken, Alexandra Brüggemann, Konstantin Mende, Maximilian Lerchenberger, Markus Rentsch, Andrej Khandoga

**Affiliations:** ^a^Department of General, Visceral, Transplantation, Vascular and Thoracic Surgery, University Hospital of Munich, Ludwig-Maximilians-University of Munich, Munich, Germany; ^b^Walter-Brendel Centre for Experimental Medicine, Ludwig-Maximilians-University of Munich, Munich, Germany; ^c^Department of Pediatric Pulmonology, Allergology and Neonatology, Hannover Medical School, Hannover, Germany; ^d^Department of General, Visceral and Vascular Surgery, Main-Kinzig-Clinics, Gelnhausen, Germany

**Keywords:** Ischemia-reperfusion, Chemokine, Fractalkine, Platelets, Leukocytes

## Abstract

**Introduction:**

The chemokine fractalkine (*CX3CL1*) is critically involved in the pathophysiology of different inflammatory diseases and myocardial ischemia-reperfusion (I/R). This study aimed to analyze the role of *CX3CL1* in the activation of platelets and leukocytes during hepatic I/R.

**Methods:**

Under inhalation anesthesia, C57BL6 mice were subjected to warm hepatic I/R (90 min/240 min). The animals were pretreated either with a function-blocking anti-mouse *CX3CL1* antibody or IgG control administered systemically before ischemia. Sham-operated animals served as controls (*n* = 7 each group). The inflammatory response and sinusoidal perfusion failure were evaluated by intravital microscopy. Hepatic transaminases plasma levels and histopathological tissue damage were determined as markers of hepatocellular injury.

**Results:**

Sinusoidal perfusion failure, leukocyte recruitment to the liver, and transaminase activities were sharply increased upon I/R compared to sham-operated mice. Firm adhesion of platelets and concordantly leukocytes to endothelial cells is reduced significantly by a function-blocking anti-*CX3CL1* antibody. We demonstrate that inhibition of *CX3CL1* signaling attenuates leukocyte adhesion in the postischemic liver but does not significantly ameliorate overall perfusion failure and hepatocellular injury.

**Discussion/Conclusion:**

Our in vivo data demonstrate a mild attenuating effect of *CX3CL1* blockade on platelet and leukocyte, but not CD4+ T cell accumulation and activation in hepatic I/R injury. We report a significant effect of blocking chemokine *CX3CL1* on sinusoidal perfusion failure without considerably improving overall hepatocellular injury during early reperfusion.

## Introduction

Ischemia-reperfusion (I/R) injury causes significant morbidity and mortality in organ transplantation, trauma, and septic or hemorrhagic shock. It is one of the main reasons for early organ failure after liver transplantation. The complex cascade of I/R-induced injury includes different mechanisms of cell death and the activation of immune cells in a sterile inflammatory response. Hepatic microcirculation is considered one of the primary targets of I/R injury of the liver. The injured endothelial lining upregulates adhesion molecules, allowing the interaction of neutrophils, CD4+ T cells, and platelets [1]. The accumulation and interaction of these leukocytes initiate a multistep inflammatory cascade.

Endothelial leukocyte capture with subsequent rolling and firm adhesion is followed by extravasation into the perivascular tissue [2, 3]. Small chemoattractant proteins attract and facilitate leukocyte recruitment to the site of inflammation. Therefore, these chemokines and their receptors are effective therapeutic targets in inflammatory processes. Fractalkine (*CX3CL1*) is a chemokine of the CX3C family [4]. Increasing evidence indicates its critical involvement in the pathophysiology of different inflammatory diseases like atherosclerosis [5, 6]. The *CX3CR1* is profusely expressed on various cell types, such as circulating leukocytes [7, 8].

Interaction between endothelial *CX3CL1* and its receptor mediates chemoattraction and promotes leukocyte adhesion in an integrin-independent way [7, 9, 10]. Correspondingly, leukocytes are recruited to *CX3CL1*-coated surfaces in venules or sinusoids [9]. Interestingly, platelets, which express *CX3CR1*, are strictly required for *CX3CL1*-induced leukocyte adhesion [8]. Endothelial *CX3CL1* also induces activation of surface-adherent platelets with consecutive platelet degranulation and exposure of the adhesion molecule P-selectin. In the liver, *CX3CR1* is most abundantly expressed on Kupffer cells (KCs) [11] and endothelial cells, while hepatic stellate cells (HSCs) are the main source of *CX3CL1* [12]. In primary biliary cirrhosis, *CX3CL1* has been shown to mediate lymphocyte recruitment to the liver [13]. In this study, we test the hypothesis that blocking *CX3CL1*/*CX3CR1* axis attenuates postischemic recruitment of leukocytes, CD4+ T cells, and platelets, resulting in protection from I/R injury without increasing the risk for hemorrhage.

## Material and Methods

### Experimental Research on Vertebrates

Female 5- to 7-week-old C57BL/6 mice were used in all experiments. The mice were purchased from Charles River® (Sulzfeld, Germany) and were housed for at least 1 week in our animal facility to recover from transportation before experiments. All experiments were carried out according to German animal safety regulations and the ARRIVE guidelines.

### Surgical Procedure and Experimental Protocol

**Fig. 1:**
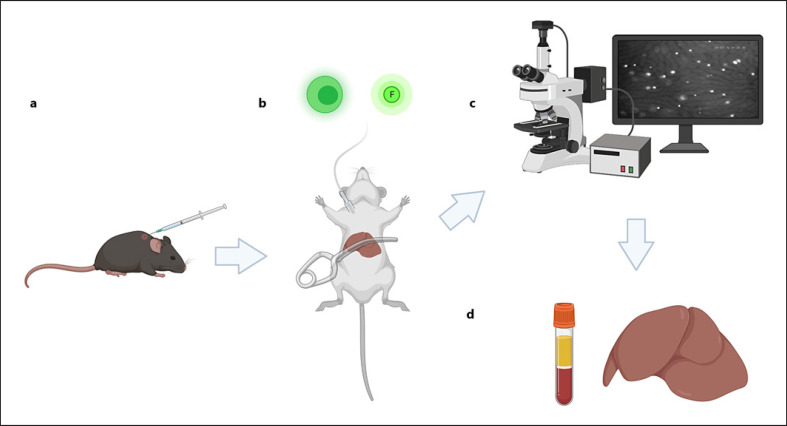
Experimental setup. **a** Female 5- to 7-week-old C57BL/6 mice were injected with function-blocking anti-*CX3CL1* monoclonal antibody TP233AF or isotype control 24 h and 30 min before ischemia. **b** Reversible ischemia of the left liver lobe was achieved by clamping the supplying nerve vessel bundle. A jugular catheter was placed for administration of saline, fluorophores, and ex vivo labeled cells from donor mice. A catheter in the ipsilateral carotid artery was used for invasive monitoring of blood pressure. **c** Modified Leitz-Orthoplan microscope was used for intravital fluorescence microscopy before collecting (**d**) the postischemic liver and whole blood for further analysis. Created with BioRender.com.

The surgical procedure was described elsewhere [14]. Briefly, under inhalation anesthesia, a microclip was used to induce a warm (37°C) reversible ischemia of the left liver lobe for 90 min by clamping the supplying nerve vessel bundle, see Figure 1. Reperfusion time was 60 min for platelets and leukocytes and 120 min for T cell experiments. A sham-operated group and four I/R groups were analyzed (*n* = 7 each): (1) sham, (2) anti-*CX3CL1* mAb + labeled platelets, (3) isotype control + labeled platelets. (4) anti-*CX3CL1* mAb + labeled CD4+ T lymphocytes, and (5) isotype control + CD4+ T lymphocytes. A function-blocking anti-mouse *CX3CL1* antibody (TP233; Torrey Pines Biolabs, Houston, TX, USA) or rabbit IgG was administered 24 h before experiments (100 μg intraperitoneal) and 10 min before reperfusion (50 μg intravenously via a jugular catheter).

### Intravital Fluorescence Microscopy

A modified Leitz-Orthoplan microscope was used for intravital fluorescence microscopy, as described previously (17). Leukocytes were stained in vivo by rhodamine 6G (0.05%, 100 μL, i.v., Sigma, Taufkirchen, Germany). Fluorescein isothiocyanate-conjugated dextran (MW 150000; 100 μL, 5%, Sigma) was used as plasma marker for sinusoidal perfusion. Platelets or CD4+ T cells were isolated from syngeneic mice with a magnetic cell separation kit according to the manufacturer's instructions and labeled ex vivo with rhodamine 6G before intravenous application and intravital microscopy. For detailed protocol of intravital microscopy, see Figure 1 and online supplementary Protocol 1 (for all online suppl. material, see www.karger.com/doi/10.1159/000524024).

### Hepatocellular Injury

Serum aspartate aminotransferase (AST) and alanine aminotransferase (ALT) activity was determined with an automated analyzer using standardized test systems, see online supplementary protocol 2. The degree of liver damage in the postischemic left lobe was assessed by a semiquantitative score described elsewhere [15] in hematoxylin/eosin stained slides.

### Statistics

ANOVA on ranks followed by the Student-Newman-Keuls test was used to estimate stochastic probability in intergroup comparison (SigmaPlot 12, Jandel Scientific, Erkrath, Germany). Mean values ± SEM are given. *p* values less than 0.05 were considered significant.

## Results

### Platelet-Endothelial Cell Interactions

**Fig. 2:**
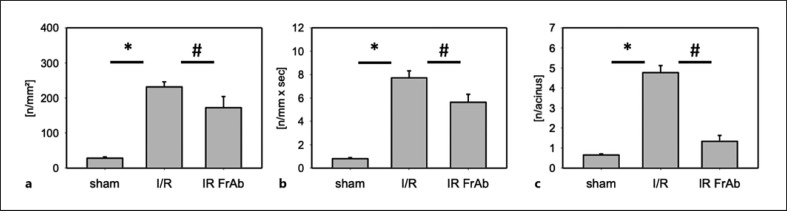
Platelet-endothelial cell interactions in vivo. Platelets in the hepatic microcirculation were labeled with Rhodamine 6G and counted via intravital fluorescence microscopy in sham-operated mouse, I/R (90 min/60 min) treated with IgG control as the vehicle and I/R after treatment with the anti-*CX3CL1* antibody TP233AF. Quantitative data on platelet-endothelial cell interactions are shown for sinusoids (**a**) and postsinusoidal venules ((**b**), rolling thrombocytes, and (**c**), adherent thrombocytes), respectively. *N* = 7 animals per group, mean ± SEM, *p < 0.05 versus sham-operated group, ^#^*p* < 0.05 versus I/R vehicle group. I/R, ischemia/reperfusion 90 min/60 min + IgG control; I/R FrAb, ischemia/reperfusion 90 min/60 min + TP233AF.

In sham-operated animals, only a few rolling or adherent platelets (0.8 ± 0.1/mm/s; 28.6 ± 3.6/mm^2^) were observed in postsinusoidal venules, whereas I/R led to a significant increase (7.7 ± 0.6/mm/s and 231.4 ± 15.7/mm^2^). Platelets were rarely observed in sinusoids of sham-operated mice (0.7 ± 0.1/acinus) but accumulated in postischemic sinusoids (4.8 ± 0.4/acinus). In contrast, pretreatment with anti-*CX3CL1* antibody significantly attenuated I/R-induced platelet-endothelial cell interactions in sinusoids (1.4 ± 0.3/acinus) and postsinusoidal venules (5.7 ± 0.5/mm/s and 176 ± 14.8/mm^2^) (Fig. 2).

### Leukocyte-Endothelial Cell Interactions

**Fig. 3:**
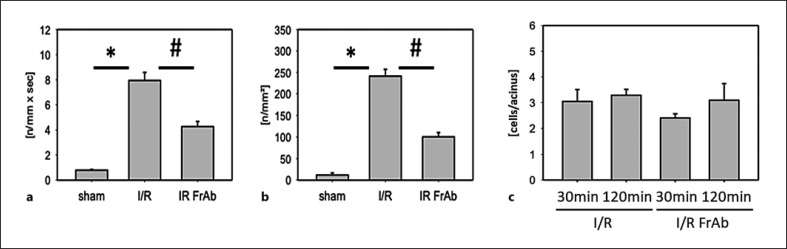
Leukocyte-endothelial cell interactions and CD4+ T cell recruitment in vivo. Leukocyte rolling and adherence were quantified using intravital microscopy in postsinusoidal venules of sham-operated mice, mice after I/R (90 min/120 min) treated with IgG control, and in mice after I/R treated with anti-*CX3CL1* antibody TP233AF. Quantitative analysis is shown for rolling (**a**) and adherent (**b**) leukocytes, respectively. CD4+ T cells in the hepatic microcirculation were labeled with CFSE and visualized via intravital fluorescence microscopy. Quantitative data on CD4+ T cell accumulation is shown per acinus (**c**). *N* = 7 animals per group, mean ± SEM, *p < 0.05 versus sham-operated group, ^#^*p* < 0.05 versus I/R vehicle group. I/R, ischemia 90 min/reperfusion 120 min + IgG control; I/R FrAb, I/R + TP233AF antibody.

Leukocyte-endothelial cell interactions were analyzed in postsinusoidal venules as an indicator of microvascular injury and hepatic inflammation after I/R. As shown in Figure 2, the numbers of rolling and firmly adherent leukocytes (1.9 ± 0.3/mm/s; 14 ± 1.4/mm^2^) in postsinusoidal venules were very low in sham-operated mice in contrast to 90 min of ischemia followed by 120 min of reperfusion in vehicle-treated mice (7.9 ± 0.7/mm/s; 241.4 ± 17.1/mm^2^). Blocking *CX3CL1* by using function-blocking anti-mouse *CX3CL1* antibody reduced the postischemic number of rolling and adherent leukocytes by about ∼20% respective ∼50% (Fig. 3), compared to the isotype IgG-treated I/R group (4.4 ± 0.5/mm/s; 105 ± 8.2/mm^2^), (data not shown).

### Migration of CD4+ T Cells

As shown in Figure 3, the number of CD4+ T cells accumulated in sinusoids did not differ between the I/R groups undergoing treatment with anti-*CX3CL1* antibody and control antibody. Similar findings were observed after both 30 min and 120 min of reperfusion.

### Sinusoidal Perfusion Failure

**Fig. 4:**
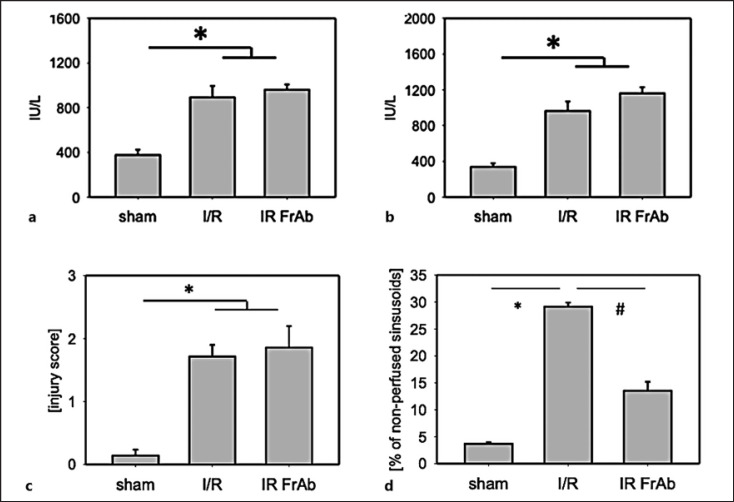
Hepatocellular injury. Serum activity of the liver enzymes AST (**a**) and ALT (**b**) was determined as a marker of hepatocellular necrotic injury. Histopathological damage was assessed in paraffin-fixed and H/E-stained tissue sections. Massive cellular damage was found after I/R (90/120 min), as indicated by the necrotic pericentral areas compared to the sham-operated mice's homogenous tissue structure. Treatment with an anti-*CX3CL1* antibody did not lead to a significant amelioration of hepatocellular damage. H/E-stained liver sections were analyzed using a semiquantitative score. Zero to three points were given for no, low, intermediate, and high tissue damage (**c**). Sinusoidal perfusion failure (= percentage of nonperfused sinusoids) was measured using intravital microscopy (**d**) as a parameter of hepatic microvascular injury. *N* = 7 animals per group, mean ± SEM, *p < 0.05 versus sham-operated group, ^#^*p* < 0.05 versus I/R vehicle group. I/R, ischemia 90 min/reperfusion 120 min + IgG control; I/R FrAb, I/R + TP233AF antibody.

Sinusoidal perfusion failure was determined using in vivo microscopy as a recognized parameter of microvascular I/R injury. In the vehicle-treated I/R group, about 29 ± 1% of all sinusoids were not perfused (Fig. 4). Anti-*CX3CL1* treatment significantly improved post-I/R perfusion failure (14 ± 2% nonperfused sinusoids).

### Hepatocellular Injury

Plasma activity of hepatic transaminases was measured as a marker of hepatocellular necrotic injury. Hepatic I/R (90 min/240 min) increased the activity of AST and ALT considerably in the vehicle-treated group compared to the sham-operated group. In contrast to the data on sinusoidal perfusion, blocking *CX3CL1* did not have a protective effect (Fig. 4).

Histopathologic analysis showed hydropic degeneration and necrotic cells after I/R compared to the sham-operated mice. Like the liver enzyme activity data, treatment with anti-*CX3CL1* antibody did not improve the postischemic tissue injury (histology score 1.9 ± 0.5 vs. 1.9 ± 0.8, Fig. 4).

## Discussion

Experimental and clinical studies suggest a crucial role of platelets in the formation of hepatic I/R injury. Thrombocytopenia markedly reduces edema formation and leukocyte infiltration in multiple acute or chronic inflammation models. Blocking the chemokine *CX3CL1* seems a promising approach to attenuate proinflammatory leukocyte recruitment and activation without compromising hemostasis.

*CX3CL1* is mainly expressed and secreted by HSCs and sinusoidal endothelium [12]. It has been reported to be involved in lymphocyte recruitment to the liver [13]. *CX3CR1* on KCs in the liver recognizes *CX3CL1* expression [11] of inflamed endothelial cells, as do activated platelets, which are consecutively recruited in large numbers to the surface of the inflamed endothelium [16].

### Platelet Recruitment to the Postischemic Liver

The initial contact with activated endothelial cells leads to platelet activation and permanent adherence to intercellular adhesion molecule 1 [1]. Fibrinogen serves as a bridging molecule [2]. Schulz et al. [16] show that *CX3CL1* on inflamed endothelium activates recruited platelets and thereby initiates leukocyte accumulation. Moreover, data from numerous cardiovascular studies suggest that the *CX3CR1*/*CX3CL1* axis affects the recruitment and activation of platelets and inflammatory monocytes to activated endothelial cells [17]. Our in vivo results demonstrate that inhibition of *CX3CL1* axis with function-blocking anti-mouse *CX3CL1* antibody significantly attenuates platelet-endothelium interactions after hepatic I/R. Both I/R-induced platelet rolling and firm adherence were reduced after treatment. These findings are consistent with the function of *CX3CL1* as a platelet-activating chemokine and explain our observation of fewer platelets being recruited to the postischemic liver. The marked reduction of platelet adhesion during early reperfusion despite hepatocellular disruption might be partly attributed to the blocked chemotactic capacities of HSCs, who are the main hepatic source of *CX3CL1* and respond rapidly to liver damage.

### Leukocyte Recruitment in Hepatic I/R

Independently of its functions in chemotaxis, *CX3CL1* directly induces capture and firm adhesion of flowing leukocytes in sinusoids and venules [9, 10]. As *CX3CL1* is expressed on endothelial cells and readily produced by HSCs, we assumed that interrupting *CX3CL1*-dependent platelet activation by function-blocking anti-mouse *CX3CL1* antibody TP233AF would also attenuate leukocyte recruitment. P-selectin contributes to the initial recruitment of leukocytes to the injury site during inflammation. However, it is not expressed on the endothelium of hepatic sinusoids, and recent data show that leukocytes adhere to liver sinusoids in a platelet-dependent manner. Platelets bind to hyaluronic acid on liver sinusoid endothelial cells via CD44 and pave the way for the adhesion of immune cells [18]. Therefore, we hypothesized that *CX3CR1*/*CX3CL1* axis can alter both the classical and nonclassical way of leukocyte adhesion. *CX3CL1*-induced P-selectin release from platelets contributes to platelet-leukocyte interaction and facilitates leukocyte adhesion [16]. Suitable for this assumption, we observe a significant decrease in leukocyte migration in the postischemic liver during early reperfusion. A possible explanation for this observation is the *CX3CL1*-induced change in the recruiting process of leukocytes. When *CX3CL1* is present, the mechanism for leukocyte adhesion is altered. The chemokine domain acts as an adhesion molecule making the association with proteoglycans and other adhesion molecules unnecessary [16]. The association between *CX3CR1* and integrins through the co-expression of *CX3CL1* and integrin ligands, such as intercellular adhesion molecule-1 and vascular adhesion molecule-1, potentiates cell adhesion even further [9].

### Migration of CD4+ T Cells to the Postischemic Liver

*CX3CL1* expressed on inflamed endothelium is thought to act as a vascular gateway for (*CX3CR1*-expressing) effector T cells by rapidly capturing them from the blood and promoting tissue migration [9]. T cells, especially CD4+ T cells, play a major role in the pathogenesis of hepatic I/R [14]. CD4+ T cells are not cytotoxic but contribute to I/R injury by modulating activation and function of other cells, such as platelets, endothelial cells, and HSCs. The ability of *CX3CL1* to act as a chemoattractant for CD4+ T cells suggests an important role in the recruitment of these cells to the postischemic liver. Moreover, allograft rejection, a process also characterized by an intense cellular immune response with an influx of circulating leukocytes into the transplanted organ [19], could be significantly reduced by treatment with *CX3CR1*-specific blocking antibodies. However, the accumulation of CD4+ T cells in the postischemic liver was not significantly affected by anti-*CX3CL1* antibody treatment. Recent findings of our group show that T cells directly interact with hepatic dendritic cells [15]. Therefore, CD4+ T cells might not depend on the chemotactic effect of *CX3CL1* in their activation, especially in the early phase of hepatic I/R analyzed in this study.

### Organ Damage and Hepatic Reperfusion Injury

We aimed to investigate whether the treatment with a chemokine inhibitor is protective against I/R injury. Such an effect seemed consequential as platelets, neutrophils, and T cells are known to mediate necrotic and apoptotic injury in the postischemic liver and play a critical role in sinusoidal perfusion failure [15, 20, 21, 22, 23]. Platelets interact with other inflammatory cells, especially neutrophils, and can directly induce tissue injury by releasing free oxygen radicals and inflammatory mediators. Our results show a significant effect of inhibition of *CX3CL1* on platelet and neutrophil activation and sinusoidal failure. However, AST/ALT levels and histopathologic damage as markers of overall hepatocellular integrity and liver necrosis were not significantly affected. The decisive mechanism why platelet and leukocyte interaction are blocked but migration and liver injury are not affected may be found in the activity of KCs. Experimental data from a murine model of liver injury shows that KCs as tissue-resident liver macrophages play an essential role in initiating and maintaining inflammatory responses. They release proinflammatory cytokines and chemokines and activate other nonparenchymal liver cells, such as endothelial or HSCs [24]. Therefore, KCs have strong detrimental or regenerative capacity in liver injury. While downregulating KC activity has been shown to be beneficial, blocking KCs obliterates their immunomodulatory function [25, 26]. As KCs strongly react to the *CX3CL1*/CX3CR-axis [11], we hypothesize that the beneficial effect of TP233AF on platelet and leukocyte recruitment is completely antagonized by the harmful blockade of KC function [27]. Another explanation for our findings is the beneficial effect of *CX3CR1*/*CX3CL1* on the inflammatory activity of dendritic cells. Sutti et al. [28] show that the *CX3CL1*/*CX3CR1* axis affects the IL-10 mediated anti-inflammatory activity of type 2 myeloid liver DCs. Moreover, blockade of *CX3CL1* reduced monocyte-derived proinflammatory DC-mediated liver damage in a hepatotoxic model of inflammatory liver injury [29]. Contrary to hepatocytes undergoing hypoxic cell death, ischemic tissue potentially destined for ischemic infarction but not irreversibly injured might be susceptible to immunomodulatory rescue and benefit from an ameliorated microperfusion during late reperfusion. Inhibiting the anti-inflammatory DC response and the regenerative capacity of KCs might cancel this road to tissue rescue. Further studies with a longer reperfusion time should be carried out to validate this hypothesis.

## Conclusion

Taken together, our in vivo data show a mild attenuating effect of *CX3CL1* blockade on platelet and leukocyte, but not CD4+ T cell accumulation and activation in hepatic I/R injury. We report a significant effect of blocking chemokine *CX3CL1* on sinusoidal perfusion failure without considerably improving overall hepatocellular injury during early reperfusion.

## Statement of Ethics

Our experiments were reviewed by the Government of Upper Bavaria before they were performed and approved as applications 8-13 and 23-13.

## Conflict of Interest Statement

The authors have no conflicts of interest to declare.

## Funding Sources

This study was supported by the German Research Foundation DFG (KH92-3/1).

## Author Contributions

D.F.: concept and design, experiments and procedures, and writing of the article; A.B.: experiments and procedures and writing of the article; K.M.: concept and design, and experiments and procedures; M.L.: concept and design and writing of the article; M.R.: concept and design; A.K.: concept and design and writing of the article.

## Data Availability Statement

All data generated or analyzed during this study are included in this article and/or its supplementary material files. Further inquiries can be directed to the corresponding author.

## Supplementary Material

Supplementary data

## Notes

Dominik Funken and Alexandra Brüggemann contributed equally.
