# Supplementary material for: Blockage of CX3CL1 Attenuates Platelet and Leukocyte Recruitment in Murine Hepatic I/R
Source: Eur Surg Res. 2022 Mar 11;63(4):244–51. doi: 10.1159/000524024 (PMC9808741; doi:10.1159/000524024)
Supplement: Supplementary file 1 — Supplementary data [file esr-0063-0244-s01.docx]

**Supplement**

**Protocol 1 Surgical procedure**

Under inhalation anesthesia with isoflurane-N_2_O + buprenorphine (0,1mg/kg body weight s.c.), two polypropylene catheters were inserted into the left carotid artery in a retrograde direction for measurement of mean arterial pressure and in the ipsilateral jugular vein for the administration of isotonic saline solution and application of fluorescence dyes. A microclip was used to induce a warm (37°C) reversible ischemia of the left liver lobe for 90min by clamping the supplying nerve vessel bundle. Reperfusion time was 60min for platelets and leukocytes and 120min for T cell experiments based on the optimal time-point for analysis of the respective cells based on previous studies of our lab.

**Protocol 2 Intravital Fluorescence Microscopy**

A modified Leitz-Orthoplan microscope was used for intravital fluorescence microscopy. Leukocytes were stained in vivo by rhodamine 6G (0.05%, 100µl, i.v., Sigma, Taufkirchen, Germany) and visualized in hepatic postsinusoidal venules using intravital fluorescence microscopy as described previously. After that, the plasma marker fluorescein isothiocyanate (FITC)-conjugated dextran (MW 150000; 100µl, 5%, Sigma) was administered, and sinusoidal perfusion was analyzed using an I2/3 filter block in sinusoids within 6-9 acini. Intravital microscopy was performed after 60min of reperfusion (platelets, leukocytes ~~neutrophils~~) or after 30min and 120min of reperfusion (CD4+ T cells) and lasted approximately 20min.

Platelets were isolated from syngeneic mice and labeled ex vivo with rhodamine 6G. Their interactions with the hepatic endothelium were analyzed for rolling and adherent platelets within sinusoids and postsinusoidal venules, respectively. A total of 1 x 10^8^ CFSE-labeled CD4+ T cells were resuspended n 200µl PBS and slowly infused intravenously via the jugular catheter after 30min of reperfusion.

Spleens of syngeneic mice were used to isolate CD4+ T cells using a bead-based magnetic cell sorting system according to the manufacturer's instructions. The purity of the CD4+ T cell purity was routinely determined by flow cytometry and above 95%. After the isolation procedure, approximately 94% of the CD4+ T cells were viable. Isolated CD4+ T cells were labeled ex vivo with the fluorescent dye carboxyfluorescein diacetate succinimidyl ester (CFSE). A total of 1 x ${10}^{7}$ CFSE-labeled CD4+ T cells were resuspended n 200µl PBS and slowly infused intravenously via the jugular catheter after 30min of reperfusion.

Capimage® software (Zeintl, Heidelberg, Germany) was used to analyze all videotaped images quantitatively. Leukocytes crossing an imaginary perpendicular through the vessel significantly slower than the centerline velocity were defined as rolling cells. Leukocyte numbers are given as cells per second per vessel cross-section. Leukocytes or CD4+ T cells firmly attached to the endothelium for more than 20s were counted as permanently adherent cells. For leukocyte quantification, the number of cells per square millimeter, the endothelial surface was calculated from the diameter and length of the vessel segment observed. T cells were quantified as the number of cells per acinus. The sinusoidal perfusion failure was calculated as the percentage of non-perfused sinusoids of all sinusoids visible.

**Protocol 3 Liver Enzymes**

Blood samples were taken from the carotid artery at the end of the experiment, immediately centrifuged at 2000xg for 10 min, and stored at -80°C. Serum aspartate aminotransferase (AST) and alanine aminotransferase (ALT) activities were determined at 37°C with an automated analyzer (Hitachi 917, Roche-Boehringer, Mannheim, Germany) using standardized test systems (HiCo GOT and HiCo GPT, Roche-Boehringer).
